# Dental measurements and Bolton index reliability and accuracy obtained from 2D digital, 3D segmented CBCT, and 3d intraoral laser scanner

**DOI:** 10.4317/jced.54428

**Published:** 2017-12-01

**Authors:** Verónica San José, Carlos Bellot-Arcís, Beatriz Tarazona, Natalia Zamora, Manuel O Lagravère, Vanessa Paredes-Gallardo

**Affiliations:** 1 Grado en Odontología [equivalent to BDS], Specialist Master of Orthodontics, Department of Stomatology, Faculty of Medicine and Dentistry, University of Valencia (Spain); 2 Assistant Lecturer, Department of Stomatology, Orthodontics Teaching Unit, Faculty of Medicine and Dentistry, University of Valencia (Spain); 3 Associate lecturer, Department of Stomatology, Orthodontics Teaching Unit, Faculty of Medicine and Dentistry, University of Valencia (Spain); 4 Associate lecturer, Department of Stomatology, Orthodontics Teaching Unit, Faculty of Medicine and Dentistry, University of Valencia (Spain); 5 Assistant Professor, PhD Orthodontics Orthodontic Graduate Program, University of Alberta, Edmonton (Canada); 6 Associate Professor. Department of Stomatology, Orthodontics Teaching Unit, Faculty of Medicine and Dentistry, University of Valencia (Spain)

## Abstract

**Background:**

To compare the reliability and accuracy of direct and indirect dental measurements derived from two types of 3D virtual models: generated by intraoral laser scanning (ILS) and segmented cone beam computed tomography (CBCT), comparing these with a 2D digital model.

**Material and Methods:**

One hundred patients were selected. All patients’ records included initial plaster models, an intraoral scan and a CBCT. Patients´ dental arches were scanned with the iTero® intraoral scanner while the CBCTs were segmented to create three-dimensional models. To obtain 2D digital models, plaster models were scanned using a conventional 2D scanner. When digital models had been obtained using these three methods, direct dental measurements were measured and indirect measurements were calculated. Differences between methods were assessed by means of paired t-tests and regression models. Intra and inter-observer error were analyzed using Dahlberg´s d and coefficients of variation.

**Results:**

Intraobserver and interobserver error for the ILS model was less than 0.44 mm while for segmented CBCT models, the error was less than 0.97 mm. ILS models provided statistically and clinically acceptable accuracy for all dental measurements, while CBCT models showed a tendency to underestimate measurements in the lower arch, although within the limits of clinical acceptability.

**Conclusions:**

ILS and CBCT segmented models are both reliable and accurate for dental measurements. Integration of ILS with CBCT scans would get dental and skeletal information altogether.

** Key words:**CBCT, intraoral laser scanner, 2D digital models, 3D models, dental measurements, reliability.

## Introduction

In orthodontics, plaster models are one of the important diagnostic tools used. Unfortunately, these models present several drawbacks such as cost, time, storage and inability to access them from other locations (1). With the development of new technologies, these drawbacks can now be eliminated. These new technologies can now digitize plaster models or teeth and help in the orthodontic diagnosis (2,3). These digital models have come to be regarded as a clinically acceptable alternative to traditional plaster models (4). Several techniques are available to generate digital three-dimensional (3D) models ranging from cone beam computed tomography (CBCT)(5-14), intraoral laser scanning (ILS) (10,15,16) or scanning of the plaster models (8,11-13,16,17).

 CBCT was introduced in the early 2000s and has been accompanied by the need to evaluate its reliability and accuracy and to ensure error predictability (5). Several authors (7,8,14) have compared measurements taken from segmented models generated by CBCT with the traditional models scanned in two dimensions (2D), obtaining sufficiently similar measurements. In another study (11), authors took measurements from plaster models, CBCTs images and laser scanned models showing that the values obtained from the three different methods were highly correlated. Similarly, some investigations have compared the reliability and accuracy of CBCT and 2D models for calculating the Bolton index (10,12,18) finding statistically significant differences but which were clinically insignificant.

The use of CBCT in orthodontics is still limited to special circumstances because of the high doses of radiation even though it offers additional dental and skeletal information. Dental information does not have much accuracy, so as an alternative, ILS generates highly accurate 3D dental models and one-to-one diagnostic information without the need for taking impressions but does not give additional skeletal information. Studies made on skulls found that ILS models produce interchangeable clinical results with CBCT models (13,17), however, one of this study (17) found statistically significant differences between both models while the other (13) found that iTero® scanner models had slightly higher correlations than CBCT models.

Regarding studies with patients, one study (15) compared traditional plaster casts and ILS models finding only slight differences that were not clinically detectable. Contrarily, other investigation (16) concluded that intraoral scanning with the iTero® scanner was less precise than extraoral cast scanning. On the other hand, Wiranto *et al.* (10) compared traditional plaster models with intraoral scanning and CBCT scanning of alginate impressions concluding that the 3D digital techniques were valid, reliable, and reproducible.

To date no studies have compared ILS and segmented CBCT models together in the same study. So the aim of the present inves-tigation was to compare the reliability and accuracy of direct (mesiodistal tooth sizes, intercanine width, intermolar width, arch length) and indirect dental measurements (Bolton index and arch discrepancy) derived from two types of 3D virtual models: generated by intraoral laser scanning (ILS) and segmented cone beam computed tomography (CBCT), comparing these with a 2D digital model (‘gold standard’).

## Material and Methods

The study design was approved by the Ethics Committee for Human Research at the University of Valencia, Spain (H1393275359870). Rights were protected by the Institutional Review Board. This study followed criteria established by the Helsinki declaration for research involving human subjects, and also conformed to STROBE guidelines.

Power analysis showed that a sample size of at least 80 patients would provide an 80% probability of detecting a difference of 0.125 mm between measurements made by two methods applying a t-paired test at a confidence level of 95%.

Two hundred and forty patients who received treatment in the Orthodontic Department between January 2014 and April 2015 were selected to take part in the study.

Previously established inclusion criteria were: (1) Patients with permanent dentition from first molar to first molar; (2) Patients whose initial records included a CBCT, an intraoral scan, and plaster models. While exclusion criteria were: (1) Tooth agenesis or extractions; (2) Presence of large restorations that could change the mesiodistal diameters of the teeth; (3) Teeth with anomalous shapes; (4) Infraoccluded teeth.

After the inclusion and exclusion criteria were applied, 100 patients were selected; 47 males and 53 females and the mean age was 25.3 ± SD 3.5 years (range 21.6 to 29.2 years).

**Figure 1:**
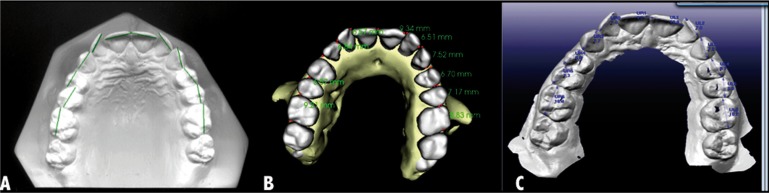
Measurement of mesiodistal tooth sizes: 2D digital (A), CBCT (B) and ILS models (C).

• A 2D digital method designed and validated at the University was used as a ‘gold standard’ measurement method, having previously confirmed the method’s accuracy and reliability for measuring plaster study models (1,19). In this method, a conventional scanner and plaster models were used to obtain a 2D image, which was stored digitally for later analysis by measurement software specific to this method (Fig. 1A). Calibration was performed before any measurement.

• The first set of 3D models evaluated were those obtained from CBCTs obtained using the Planmeca Promax 3D (Planmeca, Helsinki, Finland). Volume dimensions of 15x15x15 cm3 capture an image at a high level of detail and the voxel size was 0.4 mm. CBCT images in DICOM format were securely sent to the InVivoDental® Company website for segmentation and conversion into 3D images of the models (Fig. 1B). InVivoDental (Anatomage®; InVivoDental® Company, San Jose, CA, USA) software was posteriorly used to analyze the 3D images. InVivoDental sculpts the volume data so that the models match their original size and shape from the raw scan data.

• The second set of 3D models was obtained with an Intraoral Laser Scanner (ILS), the iTero® scanner (Align Technology, Amsterdam, Netherlands). Patients’ teeth were scanned, digitalized, and sent to Cadent iTero™for conversion into 3D models. The ILS models were posteriorly measured using OrthoCAD™ 3.0 software (Fig. 1C).

Having obtained the sample, we proceeded to undertake the tooth measurements from each of the models using the three described measuring methods. Three thousand measurements were made (30 measurements for the 100 patients) using each of the three methods, a total of 9,000 in all. The measurements that were taken were:

• Mesiodistal Tooth Sizes (TS). This size corresponds to the maximum width and distance between the mesial and distal anatomical contact points. The second and third, both upper and lower, were excluded. In badly positioned teeth, the hypothetical contact points are measured on their proximal, mesial, and distal faces.

• Intercanine distance (ICD). This is the linear distance between the cusp tips of both canines or in the centre of their wear facets should they be present, both in the upper and in the lower arch.

• Intermolar distance (IMD). This is the maximum distance between the vestibular surfaces of the first permanent molars on one and the other side of the arch, both upper and lower.

• Arch Length (AL). This is the ideal line that passes through the ideal points of contact of each of the teeth and is obtained, therefore, by joining the most mesial and distal points of each tooth selected, from the mesial of the first molar to the mesial of the upper and lower first molar. This measurement is based on a subjectively assessed ideal arch.

From these data, the following indirect measurements were calculated; Anterior (ABI) and Overall (OBI) Bolton Index (20,21), and Upper (UAD) and lower (LAD) Arch Discrepancy.

All authors contributed to the study, BT and NZ recruited participants, collected the data and compiled medical records, VS did all direct measurements, ML and VPG performed data synthesis and carried out the statistical analysis while CBA prepared the manuscript.

-Statistical analysis

All statistical analyzes were performed using a standard statistical software package (SPSS v.15.0; IBM, Armonk, NY, USA). The reliability of all measurements was analyzed by determining intraobserver and interobserver measurement error, calculated by the d-Dahlberg formula (mm) and Coefficient of Variation (CV%). The d-Dahlberg formula is a measure of absolute error and it is measured in original units, but like any absolute error, it can mean nothing if it is not relativized to the magnitude of the parameter that it is been measured. Therefore, CV has been performed as an estimator of the technical error of measurement or relative error and it has been calculated as it follows: CV= dx100/average of the parameter.

To estimate intraobserver error, the main observer took a second set of direct measurements from 40 randomly selected patients, one week after the first measurements, a total of 3,600 new measurements (30 measurements for the 40 patients) for the three methods. To estimate interobserver error, a second trained observer took direct dental measurements from the same 40 patients (a total of 3, 600 new measurements).

Data from all samples for the three measurement methods were checked using the Kolmogorov-Smirnov test to determine whether or not they presented a normal distribution; normal distribution was confirmed (*p* > 0.05).

Discrepancies between the three methods were identified by calculating differences between mean values for each measurement taken using each of the three methods. The paired Student’s t test was used to compare mean values since the objective of the study was to compare ILS versus 2D and CBCT versus 2D, and not the 3 methods themselves, in that case, repeated measurement ANOVA having 3 different methods would have been performed.

To assess concordance between methods, correlations for involved measurements were determined using Pearson’s correlation coefficients, with estimations of the slope and ordinate at the origin and their respective 95% confidence intervals by means of linear regression analysis.

## Results

Intraobserver and interobserver errors for ILS and segmented CBCT models were calculated and are shown in  Table 1. Intraobserver errors for the ILS models obtained Dahlberg d values of less than 0.31 mm for tooth measurement and less than 0.30 mm for arch measurement, while the highest CV was 1.72% for tooth size and 1.41% for arch measurement. ILS model interobserver errors presented a greater degree of error with Dahlberg d values of less than 0.44 mm for tooth measurement and less than 0.38 mm for arch measurement, while the highest CV was 1.93% for tooth size and 1.79% for arch measurement. For segmented CBCT models, error was slightly greater than with ILS. Dahlberg d values obtained for intraobserver error was less than 0.78 mm for tooth measurement and less than 0.54 mm for arch measurement, while the highest CV was 1.94% for tooth size and 1.61% for arch measurement. Interobserver errors also presented greater errors, with Dahlberg d values of less than 0.97 mm for tooth measurement and less than 0.64 mm for arch measurement, while the highest CV was 2.11% for tooth size and 1.64% for arch measurement.

**Table 1:**
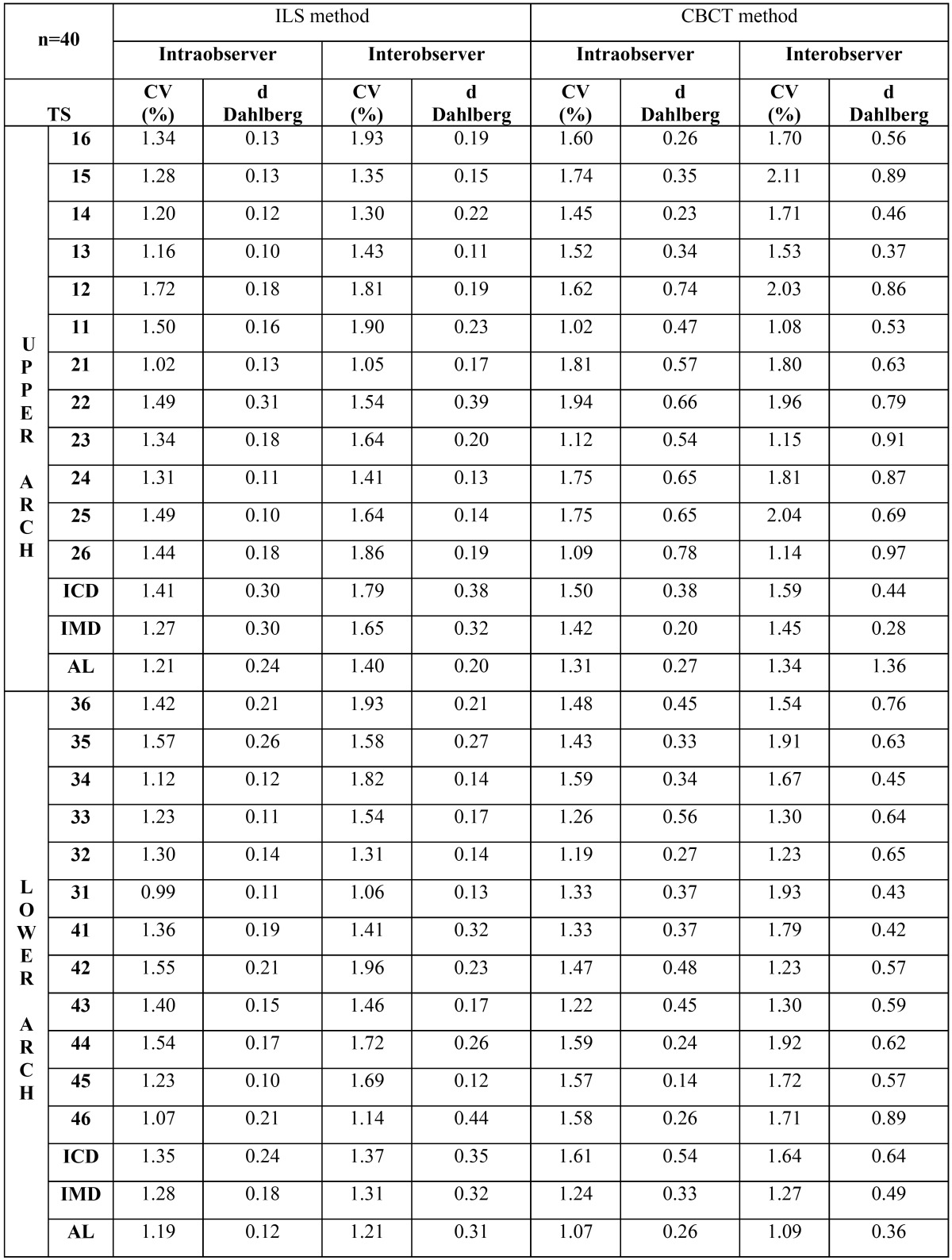
Intra and interobserver measurement errors measured by Intraoral Laser Scanner (ILS) and Cone Beam Computed Tomography (CBCT) models, calculated by Variation Coefficient (CV%) and d Dahlberg’s formula. Tooth size (TS), Intercanine distance (ICD), intermolar distance (IMD), and arch length (AL).

 Table 2 shows mean differences, standard deviations, and p-values of direct and indirect dental measurements comparing the ‘gold standard’ 2D digital method with the two 3D models: 2D digital vs CBCT and 2D digital vs ILS. No statistically significant differences were found between the 2D digital and ILS models with the highest difference being 0.77±2.47 mm; whereas between 2D digital and segmented CBCT models, 13 out of 30 differences were statistically significant, especially in the lower arch with the highest difference being 0.49±0.38 mm. Anterior and overall Bolton index results had no statistically significant differences between the three models in this study with the highest difference being 0.55±0.34% between 2D digital vs CBCT.

**Table 2:**
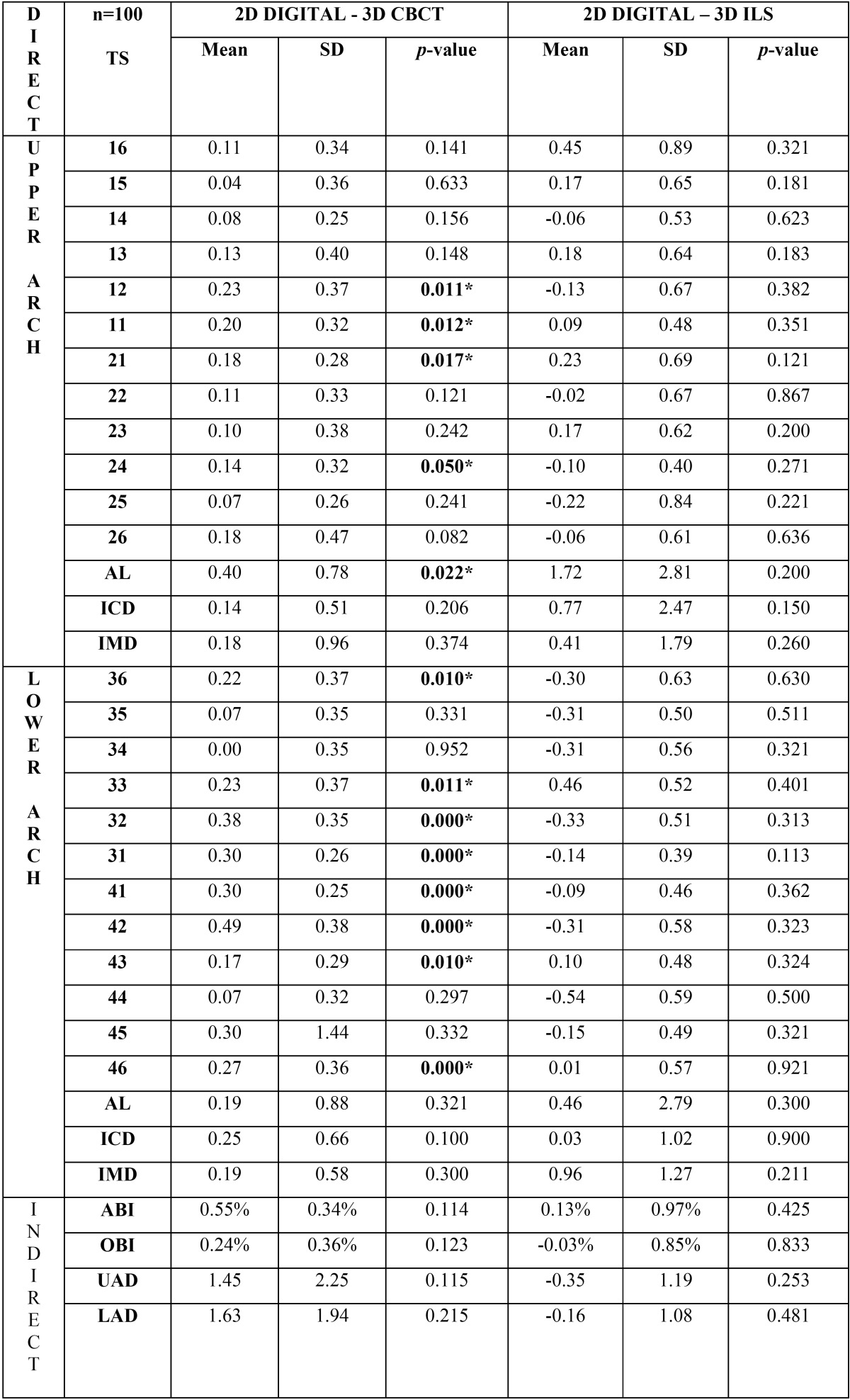
Mean differences (mm), SD and p-values in the determination of direct measurements; Tooth size (TS), Intercanine distance (ICD), intermolar distance (IMD), arch length (AL) and of Indirect measurements and indirect measurements; Anterior Bolton Index (ABI%), Overall Bolton Index (OBI%), Upper Arch Discrepancy (UAD) and Lower Arch Discrepancy (LAD) between: 2D Digital vs Cone Beam Computed Tomography (CBCT) models and 2D Digital vs Intraoral Laser Scanner (ILS) models. Significant differences* *p* < 0.05.

**Figure 2:**
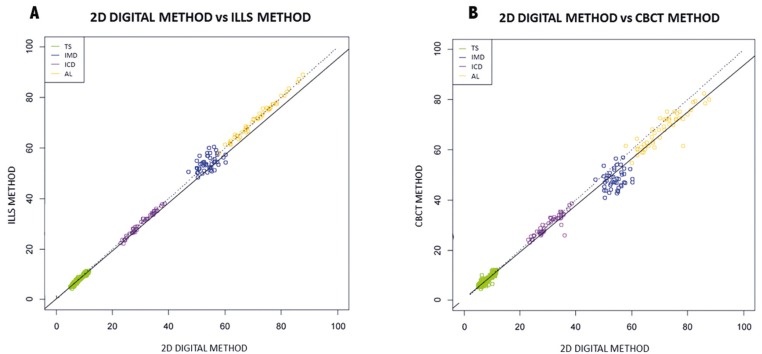
Dispersion diagram; A= Ordinates (ILS Method) vs. abscissae (2D digital method); B= Ordinates (CBCT Method) vs. abscissae (2D). Mesiodistal tooth sizes (TD; green), intercanine width (ICD; purple), intermolar width (IMD; blue), and arch length (AL; orange).

Regression analysis was performed (as shown in Fig. 2) to demonstrate the similarity between the results obtained by the three measuring methods. To assess concordance between methods, correlations for involved measurements were determined using Pearson’s correlation coefficients, with estimations of the slope and ordinate at the origin and their respective 95% confidence intervals, by means of linear regression analysis between measurements of two methods.

ILS models were more accurate for tooth sizes than segmented CBCT models. The adjustment lines comparing the two models with the ‘gold standard’ show that all of the analyzed measurements were close to the bisection and that the estimated line perfectly overlapped the main diagonal.

 Table 3 shows slopes and ordinates at origin (95% CI) and correlation coefficients calculated by regression analysis of the two methods and the 2D digital ‘gold standard’ method. All ordinate confidences contain 0 and confidence levels of the slope contain 1. This ensures that there are no systematic differences in the measurements (which would occur if the confidence level of the ordinate did not contain 0) and that an increase in the size of the object measured would represent the same increase with the two measurement methods (which would not occur if the confidence level of the slope did not contain 1). Both 3D models fulfilled these requirements for both the direct and indirect measurements.

**Table 3:**
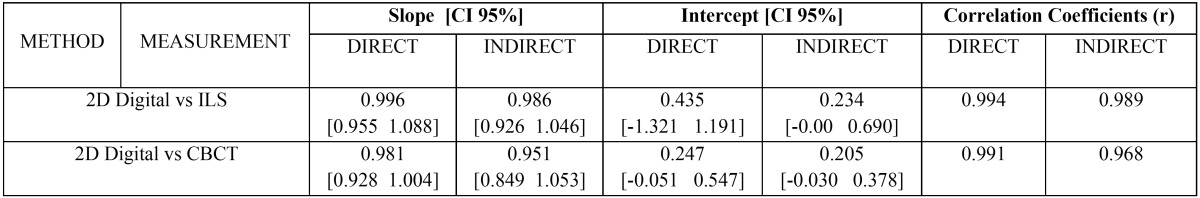
Slope and ordinate at origin, 95% Confidence Intervals (CI), and correlation coefficients for the regression analysis (r Pearson) for direct and indirect measurements between: 2D Digital vs Cone Beam Computed Tomography (CBCT) and 2D Digital vs Intraoral Laser Scanner (ILS).

## Discussion

To date, no other investigation has compared the reliability and accuracy of ILS and segmented CBCT 3D models together in the same study since it has been done separately for the ILS models (15,16), for segmented CBCT models (7,8,14) or for the ILS models and CBCT scans of alginate impressions (10). These previous three studies (7,8,14), used the InVivoDental® Company website for segmentation and conversion into 3D images of the models as our study.

Unlike most studies of this type that have taken traditional plaster models as a gold standard, the present study used 2D digital models, which have been shown to be as accurate and reproducible and with more advantages such lower cost, less time, less storage and ability to access them from other locations (1).

All the CBCTs used in this study formed part of the patients’ dental records and had been taken for other purposes (implants, third molar surgery,etc.) rather than specifically for the study. Undoubtedly, the major advantage of CBCT technology over 2D digital models is that the technique offers a wider range of diagnostic skeletal information (8). In the same way, the second set of 3D models obtained with the ILS had been taken for other purposes rather than specifically for the study (aligner orthodontic treatment etc.).

The first part of the study analyzed the reliability of the ILS and CBCT models, intra and inter-observer reliability were very high and high respectively for ILS models, and very good and good respectively for CBCT models being similar to other authors results (7,13,15).

The second part of the study calculated the accuracy of dental measurements taken from ILS and segmented CBCT models. Each of the 3D models was compared with the 2D digital models (gold standard). Unlike previous studies (11,12), the present study used segmented 3D models from CBCT since they have been shown to be more accurate in measuring tooth sizes than direct measurements on the axial cuts from the CBCT images (14). It is worth noting that the accuracy of the axial cuts from the CBCT images will vary depending on the program and CBCT apparatus used and the accuracy of the results will also depend on the orthodontist’s training and proficiency. The disadvantages of the InVivoDental® program, which provides segmentations from CBCT images to obtain 3D digital models, are that it is costly, a time consuming process as the outsourcing process is not immediate and creates company dependence since the clinicians cannot do the segmentation themselves.

Differing results between 2D digital and segmented CBCT models showed that CBCT models have a tendency to underestimate tooth sizes as all the differences were positive in favor of 2D, with greater differences in the lower arch. Nevertheless, the differences were clinically acceptable (dental differences ≤ 0.77±2.47 mm and Bolton index differences ≤ 0.55%), and could be due to the rounded interproximal contacts in the segmented CBCT models, that made dental measurement more difficult to measure and slightly lower. The present results match other author results (8,10,14), and differed from Lightheart *et al.* (6), Tarazona *et al.* (7), El-Zanaty *et al.* (9), Kim *et al.* (11), who identified statistically significant differences, but they did not find that CBCT models had a tendency to underestimate tooth sizes.

For indirect measurements, arch discrepancy results can just be compared with the Akyalcin *et al.* (13), study. On the other hand, there were no significant differences for the Bolton Index between the three methods, unlike Kim and Lagravère (11), who found statistically significant differences in the anterior Bolton Index or Wiranto *et al.* (10), and Tarazona *et al.* (18), who found differences which were clinically acceptable. Differences found in the Kim and Lagravère (11) study were within 0.3 mm while in Tarazona *et al.* (18), found differences of 0.15% for the anterior Bolton Index, and even lower than 0.06% for the Overall Bolton Index.

Comparing 2D digital with ILS models, no statistically significant differences were found agreeing with Wiranto *et al.* (10), and Cuperus *et al.* (17), but differing from Naidu and Freer (15). Unlike the present findings, Flügge *et al.* (16), found that intraoral scanning with the iTero® scanner was less accurate than extraoral model scanning with the same scanner, suggesting that intraoral conditions such as saliva or spatial limitations contributed to inaccuracy, although these limitations were not observed in the present study. Our results also agree with Akyalcin *et al.* (13), who concluded that iTero® scanner models had slightly higher correlations that CBCT models. No statistically significant differences were found in the Bolton index results, unlike Wiranto *et al.* (10), who identified differences, although these were clinically insignificant. Possible reasons for these differences could be that there is no physical barrier to the placement of measurement points with virtual models, the difficulty in scanning contact points resulting in small amounts of missing data, the numerous visualization features that depend on the orthodontist’s training and proficiency, the shrinkage of the alginate impressions and a learning curve with the software (15).

Assessing the 3D models, CBCT and ILS both presented clinically acceptable accuracy, although measurements taken from CBCT models presented statistically significant differences in comparison to the 2D models. The use of different intraoral and extraoral scanners and CBCT apparatus and programs creates some difficulty when it comes to comparing the results of different studies. In the present case, intraoral scanning eliminated possible errors arising from scanning alginate impressions or plaster models; the technique also offers the advantages of simplicity and speed. The ideal situation for getting no statistically significant difference between any measurement would be to integrate intraoral laser models with CBCT scans in order to combine the advantages of the two diagnostic techniques and get dental and skeletal information altogether.

## Conclusions

Data generated by taking measurements from ILS and CBCT models show good intra-observer and inter-observer reliability. ILS models provided statistically and clinically acceptable accuracy for all direct and indirect dental measurements, while CBCT models showed a tendency to underestimate measurements in the lower arch, although within the limits of clinical acceptability.
